# When Is the Brain Dead? Living-Like Electrophysiological Responses and Photon Emissions from Applications of Neurotransmitters in Fixed Post-Mortem Human Brains

**DOI:** 10.1371/journal.pone.0167231

**Published:** 2016-12-01

**Authors:** Nicolas Rouleau, Nirosha J. Murugan, Lucas W. E. Tessaro, Justin N. Costa, Michael A. Persinger

**Affiliations:** 1 Biomolecular Sciences Program, Laurentian University, Sudbury, Ontario, Canada; 2 Behavioural Neuroscience Program, Laurentian University, Sudbury, Ontario, Canada; 3 Human Studies Program, Laurentian University, Sudbury, Ontario, Canada; 4 Department of Biology, Laurentian University, Sudbury, Ontario, Canada; Hospital for Sick Children, CANADA

## Abstract

The structure of the post-mortem human brain can be preserved by immersing the organ within a fixative solution. Once the brain is perfused, cellular and histological features are maintained over extended periods of time. However, functions of the human brain are not assumed to be preserved beyond death and subsequent chemical fixation. Here we present a series of experiments which, together, refute this assumption. Instead, we suggest that chemical preservation of brain structure results in some retained functional capacity. Patterns similar to the living condition were elicited by chemical and electrical probes within coronal and sagittal sections of human temporal lobe structures that had been maintained in ethanol-formalin-acetic acid. This was inferred by a reliable modulation of frequency-dependent microvolt fluctuations. These weak microvolt fluctuations were enhanced by receptor-specific agonists and their precursors (i.e., nicotine, 5-HTP, and L-glutamic acid) as well as attenuated by receptor-antagonists (i.e., ketamine). Surface injections of 10 nM nicotine enhanced theta power within the right parahippocampal gyrus without any effect upon the ipsilateral hippocampus. Glutamate-induced high-frequency power densities within the left parahippocampal gyrus were correlated with increased photon counts over the surface of the tissue. Heschl’s gyrus, a transverse convexity on which the primary auditory cortex is tonotopically represented, retained frequency-discrimination capacities in response to sweeps of weak (2μV) square-wave electrical pulses between 20 Hz and 20 kHz. Together, these results suggest that portions of the post-mortem human brain may retain latent capacities to respond with potential life-like and virtual properties.

## Introduction

The fundamental principle that integrates anatomy and physiology can be effectively summarized as “structure dictates function”. This means the functional capacities of biological substrata are determined by the chemical composition, geometry, and spatial orientation of structural subcomponents [1,2]. As the heterogeneity of structure increases within a given organ, so does the functional heterogeneity. Nowhere is this more evident than in the human brain. It can be described as a collection of partially-isolated networks which function in concert to produce consciousness, cognition, and behaviour. It also responds to its multivariate, diversely energetic environment by producing non-isotropic reflections within its micrometer and nanometer spaces. The specific spatial aggregates of these dendritic alterations result in processes that have been collectively described as memory: the representation of experience.

When structures of the brain undergo changes sufficient to terminally disrupt these functional processes and the individual is ultimately observed to lose the capacity to respond to stimuli [3], the brain is said to be clinically dead. This state has been assumed to be largely irreversible. It should be noted that the specific criteria which must be achieved in order to ascribe death to an individual are not universal and exhibit a significant degree of non-consensus [4]. The precise point beyond which the brain is no longer “living”, a threshold which remains unidentified, is perhaps less definite than has been historically assumed. Without life support systems, either endogenously in the form a cardiovascular network or exogenously in the form of mechanical aids, the brain degenerates progressively until full decomposition and dissolution. Complete loss of structure is strongly correlated with the complete loss of function. When the brain is dead and the tissue has lost its structural integrity, the individual is assumed to no longer be represented within what remains of the organ.

If, however, the brain is immersed within certain chemical solutions before degeneration and decomposition, the intricate and multiform structures of the human brain can be preserved [5–7] for decades or perhaps centuries. The gyri and sulci which define the convex and concave landscapes of the brain’s outer surface as well as the cytoarchitectural features of the cerebral cortex remain structurally distinct. The deep nuclei and surrounding tract systems remain fixed in space, unchanging in time. Though structurally intact, the functions of the brain are, however, still considered to be absent. It has been assumed that the chemical microenvironment (e.g., pH, nutrient content, ionic gradients, charge disparities, etc.) of both cells and tissues within the preserved brain must be altered to such a degree to prevent degradation that these spaces no longer represent those which underlie the cellular processes which give rise to normal human cognition and behaviour.

The principle of anatomy and physiology which describes the relationship between structure and function would hold that in the presence of structural integrity so too must there be a functional integrity. If the structure-function relationship is a physical determinant, functional capacities should scale with structural loss and vice versa. Therefore the maintenance of structure subsequent to clinical death by chemical fixation could potentially regain some basic function of the tissue to the extent to which structure and function are intimately related. Here we present lines of evidence that indicate brains preserved and maintained over 20 years in ethanol-formalin-acetic acid (EFA) [8], a chemical fixative, retain basic functions as inferred by microvolt fluctuations and paired photon emissions within the tissue. They are both reliably induced and systematically controlled by the display of electrical and chemical probes which include the basic inhibitory and excitatory neurotransmitters or their precursors. Each of these profiles exhibit dosage-dependence and magnitude dependences that are very similar to those displayed by the living human brain.

## Materials & Methods

### Tissue Samples

Human brain tissue samples fixed in EFA (72% ethanol, 18% dH_2_O, 5% acetic acid, 5% formaldehyde) were subjected to a series of experimental procedures. The aim was to elicit stimulus-response patterns characteristic of structure-function relationships observed in the living human central nervous system. Three (n = 3) caudal coronal sections and four (n = 4) hemispheric sections severed within the midline sagittal plane along the medial longitudinal fissure at the level of the corpus callosum were used throughout the course of the study. Coronal sections were selected based upon exclusionary criteria including the presence of the basilar artery, Ammon’s horn, and the parahippocampal gyrus. Of the four sagittal sections, two were left hemispheres and two were right hemispheres. All samples were originally obtained from separate full human brain specimens and were therefore independently sourced. All specimens had been obtained from anonymous donors or from accredited companies (North Carolina Biological Supplies) over 20 years ago. The brains had been stored in secure areas and handled appropriately and respectfully.

### Measurement Device: Quantitative Electroencephalography

**Fig 1:**
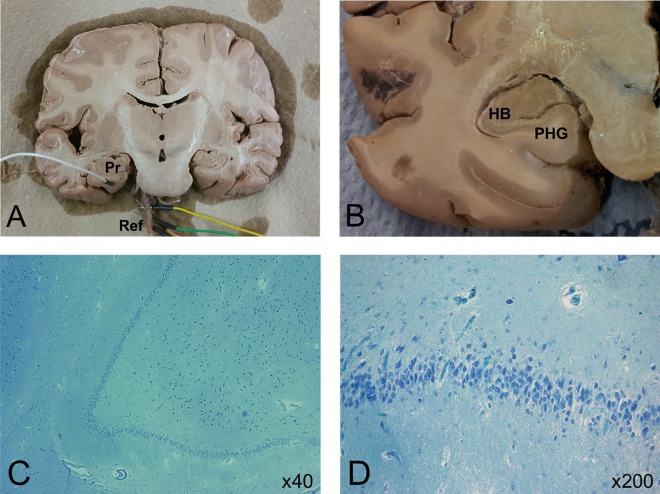
Coronal sections of human brain tissue fixed in EFA. Each section was equipped with a needle electrode inserted into the grey matter of the left parahippocampal gyrus (Pr) referenced (Ref) to the basilar artery (A). The hippocampal body (HB) and parahippocampal gyrus (PHG) served as the regions of interest (B). Cytoarchitecture of the hippocampal body fixed in EFA can be visualized under x40 (C) and x200 (D) magnification in stained (Toluidine Blue-O) sections.

A Mitsar quantitative electroencephalography (QEEG) amplifier was equipped with needle electrodes which were inserted directly into the brain tissue (Fig 1). Weak microvolt fluctuations (μV) were measured within WinEEG software throughout the course of all experiments outlined here using an HP ENVY laptop computer running Windows 8. Each experiment involved specific needle electrode placement and referencing procedures which were contingent upon the type of sample. In all cases, notch filters were applied to exclude voltage fluctuations whose frequency spectra were sourced between 50 and 70 Hz as well as 110 and 130 Hz in order to reduce environmental noise. Low and high cut filters of 1.6Hz and 50Hz were applied. Electrode impedance was regulated to <5kΩ. Data were extracted as power densities (PDs) in 30 second segments with 2 second epochs. Each extraction consisted of power measures within delta (1.5Hz– 4Hz), theta (4Hz– 7.5Hz), alpha (7.5Hz– 14Hz), beta1 (14Hz– 20Hz), beta2 (20Hz– 30Hz), and gamma (30Hz– 40Hz) band ranges.

In the case of coronal sections, needle electrodes were inserted into the basilar artery which served as an electrical reference (Fig 1). A single electrode was then inserted into the region of interest. The left and right parahippocampal gyri and hippocampal bodies served as the areas of interest throughout the course of the study. Only one structure was ever probed for a given trial due to limitations of the referencing procedure. This means that it was not possible to infer in real-time whether or not hemispheric analogues or adjacent structures were simultaneously responding to the same stimulus. Once the needle electrode was inserted into the region of interest and was referenced to an average of electrodes inserted into the basilar artery, microvolt fluctuations could be recorded. Sagittal sections of fixed human brain tissue were probed similarly. The basilar artery always served as the electrical reference point. In the case of the sagittal sections, the primary loci of interest were the transverse temporal gyri. Needle electrodes were inserted directly into the transverse temporal gyri within both the postero-medial and antero-lateral subdivisions. The precise stimulation procedure carried out over the course of electrophysiological measurement is outlined elsewhere.

### Measurement Device: Photomultiplier Tube

Photon measures were obtained within a darkened chamber concurrently with QEEG for trials involving applications of glutamate to the brain. Raw photon counts were recorded using a single photomultiplier tube (PMT) that was suspended 10cm above the brain specimen. The PMT was a DM0090C model from Sens-Tech Sensor Technologies, with a spectral response range between 300–850 nm (visible light). Sens-Tech Counter timer software recorded digital output from the photomultiplier tube at a 50 Hz sampling rate for 3000 readings (20 msec data points for 60 seconds) on a Lenovo ThinkPad laptop that was positioned outside of the enclosed chamber via USB output cables. To remove the contributions from dark counts (i.e., those associated with the intrinsic photoelectric circuitry), counts measured when the brain tissue was present were subtracted from baseline conditions when no tissue was present. It was determined that the use of equipment measuring electric potential differences (QEEG) and photon counts over time would serve as a measure of internal validity, confirming the presence of systematic response patterns which could be observed by both measurement devices independently.

### Procedures

#### Chemical Application

Within the context of the living brain, chemical signals in the form of neurotransmitters are transduced at the level of the receptor into miniature inhibitory and excitatory post-synaptic potentials or IPSPs and EPSPs [9]. They undergo summation within the post-synaptic cell resulting in further propagation of electrochemical signals. The proximal cause of ionic inflow to the cell is receptor-modulated by agonistic and antagonistic ligands interacting at the level of the plasma membrane. Here, we have designed a series of experiments which involved the application of neurotransmitters, their precursors, or known modulators of receptors within the central nervous system to coronal sections of human brain tissue fixed in EFA. L-glutamic acid (glutamate), 5-Hydroxy-L-tryptophan (5-HTP), (−)-nicotine, and ketamine were obtained from Sigma-Aldrich (USA) and serially diluted into various concentrations ranging between 1 M and 1 nM.

Experiments involving the application of chemical compounds to the tissue were associated with a specific injection protocol. Each injection was preceded by washing the surface of the coronal slice with 10% ethanol-formalin-acetic acid (EFA) which was followed by a 30 second baseline condition during which electrophysiological recordings were obtained. A 1 mL aliquot of the solution was injected on to the surface of coronal slices at the parahippocampal-hippocampal interface. The point of injection was therefore crudely distributed over both regions of interest. Therefore, upon injection of the compound, any changes in microvolt fluctuations observed within either the parahippocampal gyrus or hippocampal body was not necessarily due to stimulation of the probed area alone. Instead, adjacent regions, whose efferent and afferent connections likely contributed to local activity, should be considered as potential sources of any differences in addition to the probed area.

#### Electrical Stimulation

The human primary auditory cortex is localized within the medial two-thirds of the transverse temporal gyrus or Heschl’s gyrus (HG) whereas the antero-lateral component is designated as an adjacent, non-primary region [10]. Morphometric analyses have revealed the reliable presence of tonotopic subfields along HG which run perpendicular to the classical postero-medial-to-antero-lateral cytoarchitectonic organizational divisions [11]. These “tonotopic maps” are frequency-representing gradients within the tissue which process primary auditory information received by way of the medial geniculate nucleus of the thalamus. An experimental verification of preserved frequency-discrimination within the postero-medial component of Heschl’s gyrus in chemically fixed brain specimens could support a structural-functional model in post-mortem tissue.

Square and sine wave-forms were generated using Audacity’s (2.0.5) tone-generating tool on an HP ENVY laptop running Windows 8. Each signal consisted of 30 seconds of a square or sine wave with an associated frequency of 20 Hz, 100 Hz, 500 Hz, 1 kHz, 2 kHz, 5 kHz, 10 kHz, 12 kHz, 15 kHz, or 20 kHz. We selected the 20 Hz– 20 kHz range to reflect the operating range of the human auditory pathways. Though the relationship between pressure waves and their transduced electrical equivalents is not 1:1, the large band range would accommodate our practical, methodological needs to demonstrate frequency-dependent discrimination. Amplitude of the signal within Audacity was set to 0.8 (a.u.). The signal output was regulated to 10% of maximum audio card output. A coaxial cable coupled to an electronic breadboard jumper cable by an alligator clip served as a stimulating probe which was inserted into the tissue. The voltage equivalent at the level of the needle probe positioned within ~2 mm adjacent to the stimulating probe, 2 μV, was measured directly by the electrophysiological recording device [12].

The measurement procedure involved inserting both the data collecting needle probe from the electrophysiological recording device and the stimulating probe into either the postero-medial or antero-lateral division of the right or left HG. The needle probes were separated by ~ 2 mm where the stimulating probe was always the lateral-most probe. Each trial consisted of a 30 second baseline followed by 270 seconds of stimulation. The 270 second stimulation period was further divided into 9 periods, each with an associated frequency. Frequencies were counterbalanced to eliminate order effects.

### Methods of Analysis

Power densities (PDs) were extracted from WinEEG 2.93.59 (07.2013) and imported to SPSS v19 for subsequent analysis. The spectral analysis technique and resulting power values were selected so as to isolate frequency-dependent signatures from the overall signal. Alternative signal processing techniques of raw data extractions were employed when analyzing the tissue’s response to electrical stimulation so as to infer information processing disparities as a function of the probed region. Fractal geometry, when applied to statistical analyses, refers to a method of generating a ratio which represents an index of complexity–how the detail in the pattern or signal under analysis changes with respect to the scale or level of discourse at which it is being measured and examined. The Higuchi Fractal Dimension (HFD) algorithm is one such method of determining statistical complexity and was employed in the present study involving electrical stimulation of HG as has been employed in other studies which have examined electroencephalographic complexity [13]. Additional data processing was conducted prior to the HFD analysis. It was assumed that the effect of the stimulus on the tissue as inferred by the recorded QEEG signal would be best illustrated if a difference was taken between each of the segments corresponding to the periods of frequency-specific stimulation and the baseline electrophysiological recordings from the fixed brain. Thus each of the segments underwent the following transform prior to HFD analyses:

$$ND=\left(RS_{xy}-RS_{BL}\right)$$

Where *ND* is the new data file generated, *RS*_*xy*_ is the raw signal segment for frequency *xy*, and *RS*_*BL*_ is the raw signal baseline (no frequency), for each individual trial.

## Results

### Surface Injections of Nicotine

**Fig 2:**
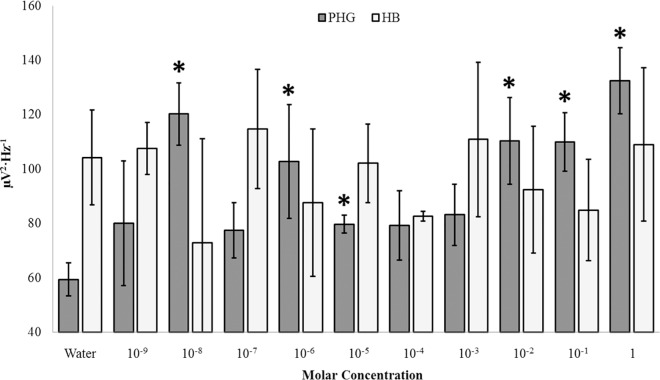
Nicotine Response. Theta (4Hz– 7.5Hz) PDs as a function of nicotine concentration within the right parahippocampal gyrus (PHG) and hippocampus (HB). Significant differences from sham (Water) after correction (α = .005) are indicated.

Analyses of variance (ANOVAs) revealed statistically significant three-way interactions between structure, hemisphere, and concentration for differences of theta (4Hz– 7.5Hz) and alpha (7.5Hz– 14Hz) PDs (S1 File). Alpha effects were weak, and were eliminated when accounting for multiple comparisons. The three-way theta power interaction was conspicuous [F(10,130) = 4.06, p < .001, η^2^ = .22]. Right parahippocampal theta power differed as a function of concentration [F(10,32) = 8.02, p < .001, η^2^ = .78] (Fig 2). The effect was dose-dependent with a general linear increase (r = .60). The effect was not present for the immediately adjacent hippocampal body (p>.05) or contralateral structures (p>.05) which was the major source of the interaction. The control (water) condition was associated with differences in power as a function of structure; however, there were no within-structure differences in theta power as a function of hemisphere (p>.05).

**Fig 3:**
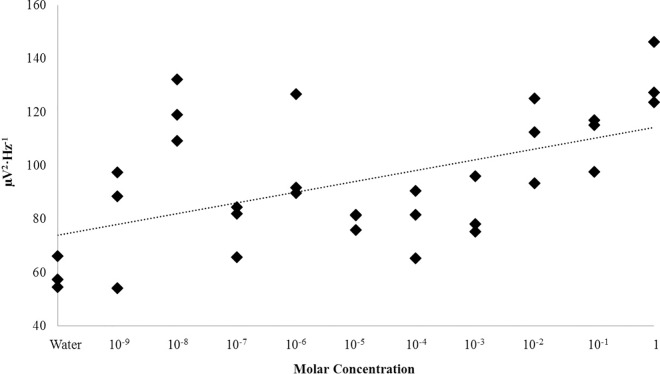
Concentration Dependence: Nicotine. Theta (4Hz– 7.5Hz) PDs within the right parahippocampal gyrus as a function of the concentration of nicotine injected over the surface of the tissue.

The primary sources of variance associated with increases in theta power within the right parahippocampal gyrus following surface injections of nicotine were between sham condition (M = 59.43, SEM = 3.49) and a number of concentrations, namely: 10 nM (M = 120.20, SEM = 6.63), 1 uM (M = 102.77, SEM = 12.03), 10 uM (M = 79.70, SEM = 1.90), 1 mM (M = 83.23, SEM = 6.48), 10 mM (M = 110.33, SEM = 9.21), 100 mM (M = 109.93, SEM = 6.19), and 1 M (M = 132.47, SEM = 7.00). Effect sizes ranged between 75% and 96%. Theta power was also observed to linearly increase as a function of concentration, r = .53, p < .001; rho = .50, p < .005 (Fig 3). This relationship was strengthened when removing trials involving the peak 10 nM concentration (r = .71, p < .001). In fact, power fluctuations were essentially non-linear below concentrations of 1 μM after which the relationship strengthened markedly. After Bonferonni correction for multiple comparison (α = .005), three concentration ranges could be discerned, each defined by a “peak” wherein the average was visually increased as seen in Fig 2. Three concentrations, 10 nM, 1 uM, and 1M, were selected across the range of the set for further experimentation where the time-course of theta fluctuations would be monitored post-injection.

**Fig 4:**
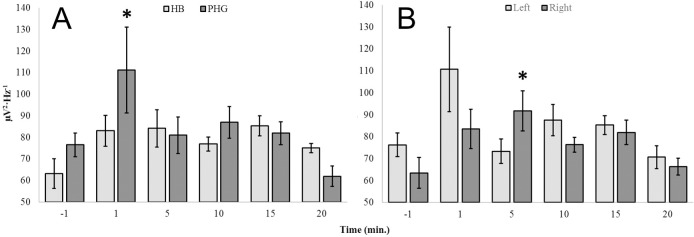
Time Dependence: Nicotine. Theta (4Hz– 7.5Hz) PDs as a function of time (min) from injection (time = 0 or between -1 and 1) of 10 nM nicotine for hippocampal (HB) and parahippocampal (PHG) loci (A) as well as between left (Left) and right (Right) hemispheres (B).

A grand mean of the “peak” concentration theta power responses to surface injections of nicotine (M = 95.06, SEM = 7.47) were increased relative to baseline conditions (M = 78.41, SEM = 3.34) [t(34) = 2.04, p = .05, r^2^ = .11]. An ANOVA revealed that 10 nM injections over the surface of the parahippocampi induced time-dependent fluctuations in theta power, F(5,35) = 2.60, p < .05, η^2^ = .30. These disparities were likely driving the differences observed with the grand-means. Other concentrations did not demonstrate robust time-dependent effects (p>.05). Homogeneous subsets identified two groups wherein the major sources of variances were between theta power 20 minutes post-injection (which was indistinguishable from baseline theta power) and the 1 minute immediately following injection (r^2^ = .27, p < .05). These differences in theta power within the PHG are visualized in Fig 4A. A clear initial increase was noted, followed by a decrease to baseline levels over time.

Further analysis of hemispheric effects indicated that theta power increased significantly within right hemispheric structures (parahippocampal gyri and hippocampal bodies) 5 minutes post-injection of 10 nM nicotine (M = 91.75, SEM = 9.08) relative to baseline conditions (M = 63.43, SEM = 7.04), t(10) = 2.46, p < .05, r^2^ = .38 (Fig 4B). The effect was specific to the theta band and did not generalize to left hemispheric structures [t(10) = .38, p>.05]. Left hemispheric structures did not demonstrate time-dependent changes relative to baseline. As is apparent in Fig 4B, the high degree of variability associated with left hemispheric theta power 1 minute post-injection may have masked an early-phase homologous effect.

**Fig 5:**
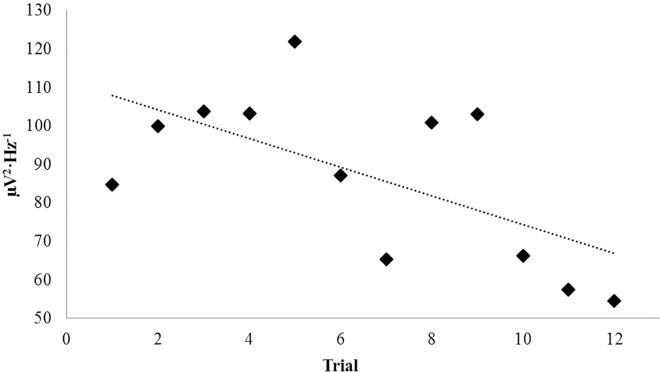
Repeated Exposure: Nicotine. Theta (4Hz– 7.5Hz) PDs upon injection of water (control) as a function of time.

PDs collected serially over several weeks of experimentation were plotted over time in order to discern any long-term effects associated with repeated and protracted exposures to surface injections of various concentrations of nicotine. Time, in this case, was represented by trial order. Investigating linear relationships between band-specific power and trial order (implicitly time) within the tissue revealed a negative correlation for the theta-band PDs during the sham condition (r = -.62, p < .05). The relationship is plotted in Fig 5. Selecting for the first and last three sham trials, a significant decrease in theta power was observed, t(4) = 5.40, p = .006, r^2^ = .88. Three conspicuous trials can be seen in Fig 5 which can be interpreted as “peaks” within the negative trend which are represented at points 5, 8 and 9. Each trial was determined to have originated from separate coronal slices (n = 3). Trials 8 and 9 were preceded by injections of 10 mM nicotine. Considering that conditions associated with each trial were randomized and order-effects were unlikely due to chance alone, this observation could be relevant.

### Surface Injections of 5-Hydroxy-L-trypotophan

**Fig 6:**
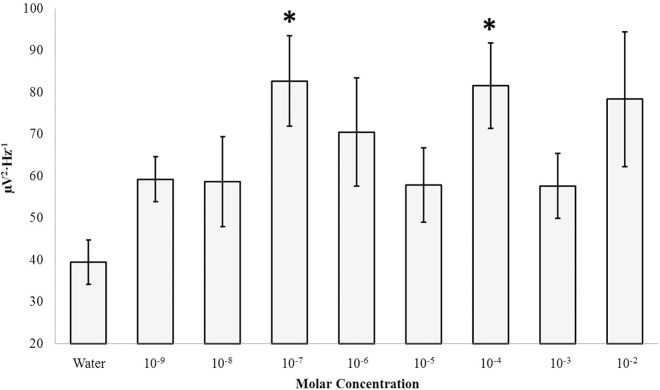
5HTP Response: Right Hippocampus. Theta (4Hz– 7.5Hz) PDs within the right hippocampal gyrus as a function of the molar concentration of 5-HTP applied to the surface of coronal sections of human brain tissue. Significant differences are indicated (p>.05).

Applications of various concentrations of 5-HTP to the coronal sections revealed a number of features (S2 File). Significant increases in theta (4Hz– 7.5Hz) PDs within the right hippocampal body were noted for the 100 nM (M = 82.67, SEM = 10.81) and 100 μM (M = 81.53, SEM = 10.21) concentrations relative to the water control (M = 39.47, SEM = 5.32) where p < .05 and effect sizes were 76% and 77% respectively (Fig 6). Other frequencies were unaffected and the contralateral hippocampal body did not demonstrate similar response patterns (p>.05).

**Fig 7:**
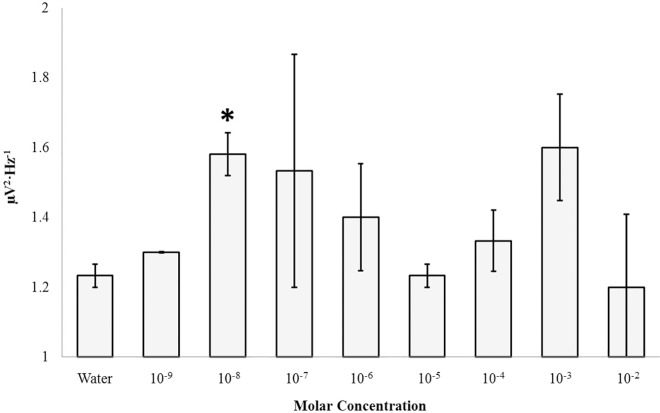
5HTP Response: Right Parahippocampal Gyrus. Gamma (30Hz– 40Hz) PDs within the right parahippocampal gyrus as a function of the molar concentration of 5-HTP applied to the surface of coronal sections of human brain tissue. Significant differences are indicated (p>.05).

The right parahippocampal gyrus displayed increased gamma (30Hz– 40Hz) activity upon injection of 10 nM 5-HTP (M = 1.58, SEM = .06) relative to water control [(M = 1.23, SEM = .03), t(4) = 4.98, p = .008, r^2^ = .86 (Fig 7)]. The contralateral parahippocampal gyrus did not express similar differences as a function of any concentrations of 5-HTP (p>.05). Other frequency-specific microvolt fluctuations remained unaffected by surface injections of 5-HTP applied to the right parahippocampal gyrus (p>.05). Dose-dependent linear relationships or changes in microvolt potentials over time could not be identified for coronal sections exposed to 5-HTP. This feature was observed for all of the other applied chemical compounds.

### Surface Injections of Glutamate

**Fig 8:**
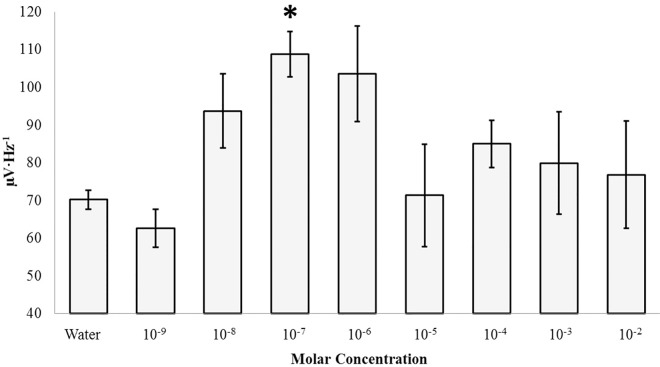
Glutamate Response. Global power (1.5Hz– 40Hz) within the left parahippocampal gyrus as a function of concentration of glutamate. A significant increase in mean global power after Bonferonni correction (α = .006) is indicated.

**Fig 9:**
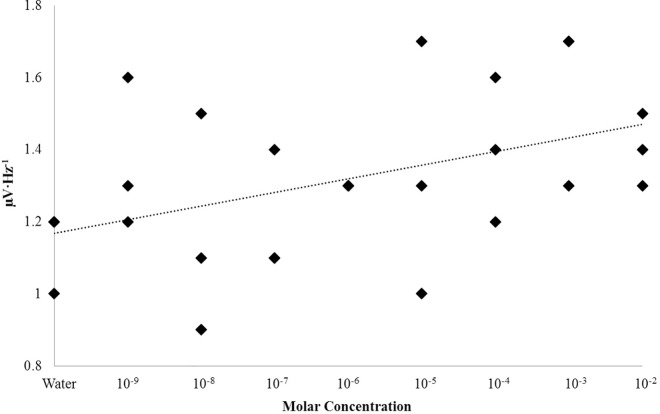
Concentration Dependence: Glutamate. Gamma (30Hz– 40Hz) power within the left parahippocampal gyrus plotted as a function of concentration of the injected material.

**Fig 10:**
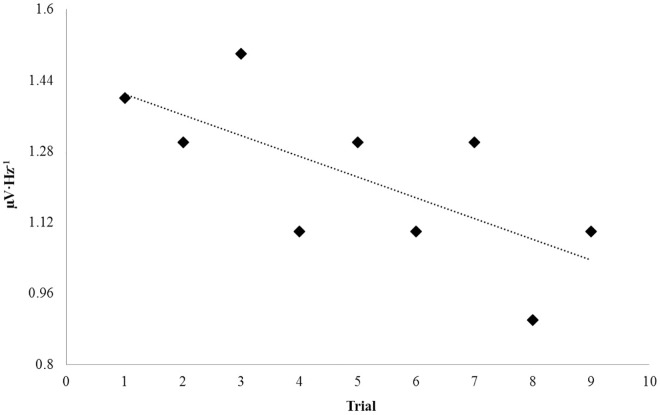
Repeated Exposure: Glutamate. Gamma (30Hz– 40Hz) power within the left parahippocampal gyrus for trials involving surface injections of 10^−8^ to 10^−6^ M glutamate as a function of trial order, or implicitly, time.

An ANOVA revealed a three-way interaction between structure, hemisphere, and concentration for global (1.5 Hz– 40 Hz) PDs [F(8, 107) = 3.02, p < .01, η^2^ = .20] (S3 File). Selecting for the left parahippocampal gyrus, global power (1.5Hz– 40Hz) increased upon injection of concentrations of 100 nM (M = 108.76, SEM = 6.07) of glutamate relative to water (sham) control [(M = 70.23, SEM = 2.49) [t(4) = -5.88, p < .005, r^2^ = .90 (Fig 8)]. This effect was largely due to increases in delta (1.5Hz– 4Hz) activity between the same conditions [t(4) = -4.99, p < .01, r^2^ = .86]. Conspicuous high-frequency modulations by nanomolar-range glutamate can be observed in Figs 9 and 10. The left parahippocampal gyrus demonstrated increased theta band PDs upon surface injections of 10 μM concentrations (M = 89.37, SEM = 1.29) of glutamate relative to the sham condition [(M = 69.20, SEM = 4.10), t(4) = -4.70, p < .01, r^2^ = .85]. Finally, increases of 0.3 to 0.5 μV·Hz^-1^ were observed for probed left parahippocampi within the gamma band upon surface injections of 10 mM and 1 mM glutamate respectively with associated effect sizes of 68% and 70%. The anatomically adjacent left hippocampal body did not display significantly different spectral power upon surface injections of any concentration of glutamate relative to the sham condition (p>.05).

A conspicuous linear relationship between gamma activity within the left parahippocampal gyrus and the concentration of the injected material was also observed [r = .45, p < .05; rho = .47, p < .05 (Fig 9)]. Linear relationships between concentration of the injected material and spectral power within any band could not be identified within alternative structures (p>.05). Selecting for concentrations between 10^−8^ to 10^−6^ M glutamate, conditions which produced the greatest magnitude shifts in computed global power relative to the water control, a strong negative correlation was observed between trial order and gamma power [r = -.73, p < .05; rho = -.71, p < .05 (Fig 10)]. This relationship indicates that gamma power linearly decreased as a function of time upon injections of the same concentrations of glutamate which transiently increased gamma power activity. This relationship was not observed when plotting trial order with gamma power independent of selective concentration bands (p>.05). In other words, the negative relationship was only maintained when narrow-band concentrations which induced maximal responses relative to control conditions were selected.

Two minor right hemispheric effects were noted. First, an increase in delta power (1.5Hz– 4Hz) within the right parahippocampal gyrus was noted upon injection of 1 mM (M = 401.97, SEM = 14.67) glutamate relative to the sham condition (M = 351.70, SEM = 9.27), t(4) = -2.90, p < .05, r^2^ = .68. When considering a computed global average of spectral power differences the effect size associated with significant differences between 1 mM and the sham condition increased to 73%. Second, an increase in gamma power (30Hz– 40Hz) within the right hippocampal body was noted upon surface injections of 10 nM (M = 1.63, SEM = .07) glutamate relative to the sham condition (M = 1.27, SEM = .09) [t(4) = 3.32, p < .05, r^2^ = .73].

**Fig 11:**
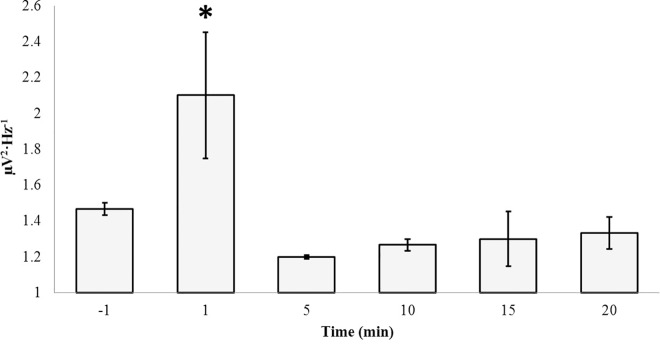
Time Dependence: Glutamate. Gamma (30Hz– 40Hz) power within the left parahippocampal gyrus as a function of time elapsed from the point of surface injections of 100 nM (10^−7^ M) glutamate.

Having identified the “peak” concentration of 10^−7^ M, which optimally induced gamma power increases within the left parahippocampal gyrus, a series of trials were completed in order to plot the time-course of glutamate power over 20 minutes (Fig 11). An ANOVA revealed significant differences in gamma power over time, F(5, 17) = 4.25, p < .05, η^2^ = .64. No other frequency band was affected (p>.05). *Post-hoc* tests revealed two homogeneous subsets with the 1 minute post-injection condition loading separately from all other time conditions. The increase in power was equivalent to ~ 0.7 μV·Hz^-1^ on average.

### Surface Injections of Ketamine

Dose-dependent curves of high-frequency PDs identified within the left parahippocampal gyrus occurred from exposure to a narrow band of molar concentrations of glutamate. We investigated the potential mechanisms governing the effects. A focused experimental procedure was designed whereby left hemispheric hippocampal bodies and parahippocampal gyri were exposed to various concentration of ketamine (S4 File), an N-methyl-D-aspartate (NMDA) receptor antagonist [14]. It was hypothesized that decreases in high-frequency PDs within the parahippocampus but not the hippocampus would result if the operating mechanism was common to that which was underlying the glutamate effects.

**Fig 12:**
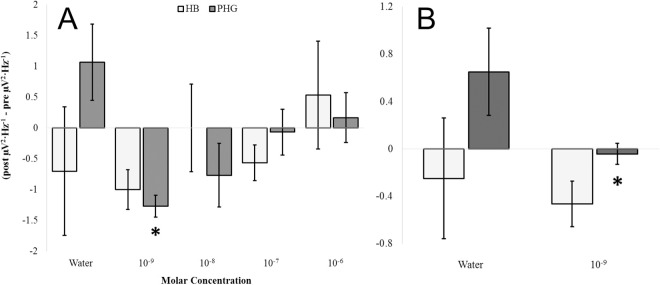
Ketamine Response. Beta1 (14Hz– 20Hz) SPD differences from the pre-injection period to the post-injection period for left hemispheric hippocampal bodies (HB) and parahippocampal gyri (PHG) exposed to various concentrations of ketamine (A). High frequency PDs computed from an average of beta1 (14Hz– 20Hz) and gamma (30Hz– 40Hz) SPD differences from the pre-injection period to the post-injection period for left hemispheric hippocampal bodies (HB) and parahippocampal gyri (PHG) exposed to 1nM ketamine compared to sham injection (B). Significant differences are indicated (p < .05).

A one-way ANOVA selecting for the parahippocampal gyrus revealed that beta1 PD differences from the pre-injection period (i.e., a 30 second period immediately preceding the injection) to the post-injection period (i.e., a 30 second period immediately following the injection) differed as a function of concentration of ketamine [F(4,14) = 4.09, p < .05, η^2^ = .62 (Fig 12A)]. Homogeneous subsets revealed the primary source of variance was a difference between the 1 nM condition and the water control (p < .05). *Post-hoc* t-tests confirmed this difference marked by a decrease in power after injection [t(4) = 3.63, p < .05, r^2^ = .77]. A proportionally similar decrease was also noted when using an average of beta1 and gamma activity [t(4) = 2.86, p < .05, r^2^ = .67 (Fig 12B)]. Concentration effects were not noted for the hippocampal body across any spectral power band (p>.05). That high-frequency activity was enhanced by glutamate and suppressed by ketamine suggests a common site of action and reduces the probability that the effects were simple artifacts of injections. That both of these phenomena were observed within the parahippocampal gyrus but not the hippocampus indicates internal consistency.

**Fig 13:**
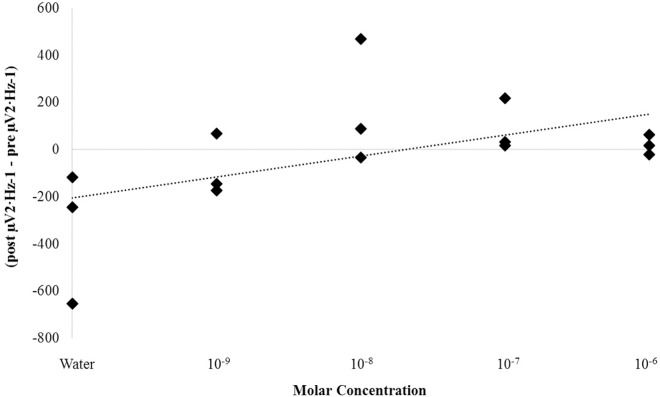
Concentration Dependence: Ketamine. Non-parametric correlation between delta (1.5Hz– 4Hz) SPD differences from the pre-injection period to the post-injection period and molar concentration of ketamine for the left parahippocampal gyrus. A significant correlation was identified (rho = .60, p < .05).

**Fig 14:**
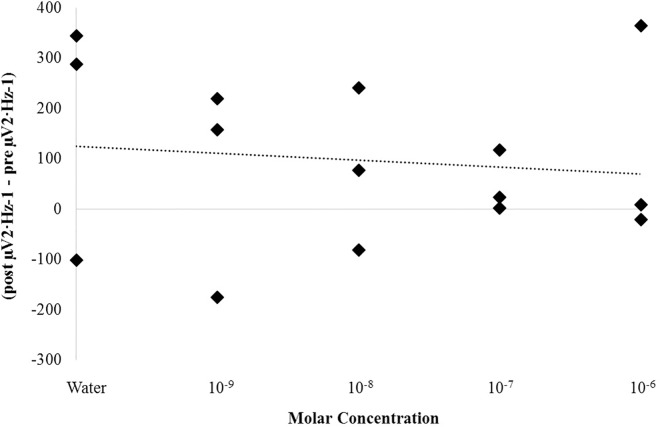
Null Hippocampal Effect: Ketamine. Non-parametric correlation between delta (1.5Hz– 4Hz) SPD differences from the pre-injection period to the post-injection period and molar concentration of ketamine for the left hippocampal body. No significant correlation was identified (p>.05).

A non-parametric correlation was identified between low-frequency (delta to alpha) but not high-frequency (beta1 to gamma) SPD differences within the left parahippocampal gyrus from pre- to post-injection periods and the molar concentration of ketamine administered (rho = .60, p < .05). These differences were primarily due to an underlying positive correlation between delta PD differences from pre- to post-injection periods and molar concentrations of ketamine, rho = .56, p < .05 (Fig 13). The adjacent hippocampal body did not demonstrate any statistically significant relationships between expressed power density differences from pre- to post-injection periods and drug concentration (p>.05). Fig 14 demonstrates the non-relationship observed between delta-band PD differences from pre- to post- injection periods and molar concentration for the hippocampal body (rho = -.10, p = .73) which was in stark contrast to that which is displayed in Fig 13 for the parahippocampal gyrus.

**Fig 15:**
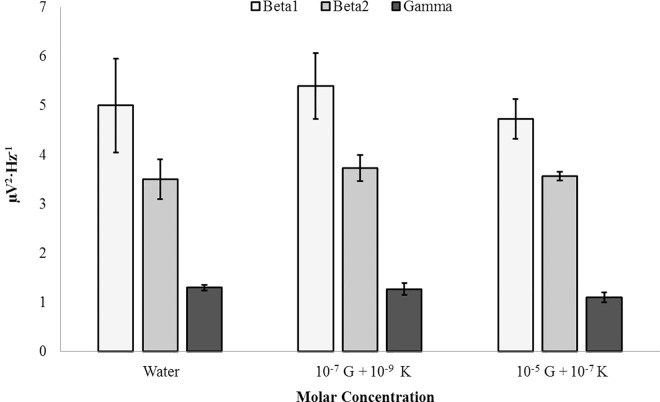
Glutamate-Ketamine Response. Beta1 (14Hz– 20Hz), beta2 (20Hz– 30Hz), gamma (30Hz– 40Hz) PDs within the left parahippocampal gyrus exposed to surface injections of water (Water), 10^−7^ M glutamate and 10^−9^ M ketamine (10^−7^ G + 10^−9^ K), or 10^−5^ M glutamate and 10^−7^ M ketamine (10^−5^ G + 10^−7^ K). No significant differences were observed (p>.05).

In order to substantiate the potential receptor-mediated mechanisms governing increases in high-frequency microvolt fluctuations associated with NMDA receptor agonist glutamate and similar decreases associated with NMDA receptor antagonist ketamine, mixed solutions were generated. Left parahippocampi were exposed to surface injections of water, a mixed solution of 10^−7^ M glutamate and 10^−9^ M ketamine (i.e. the optimal concentrations which modulated high-frequency microvolt fluctuations), or a mixed solution of 10^−5^ M glutamate and 10^−7^ M ketamine which served as an alternative control (S5 File). No significant differences between injection conditions were noted for beta1, beta2, and gamma PDs (p>.05). Fig 15 demonstrates the mutual nullification of glutamate- and ketamine-mediated high-frequency effects within the left parahippocampal gyrus.

### Glutamate-Induced Microvolt Fluctuations and Coupled Photon Emissions

**Fig 16:**
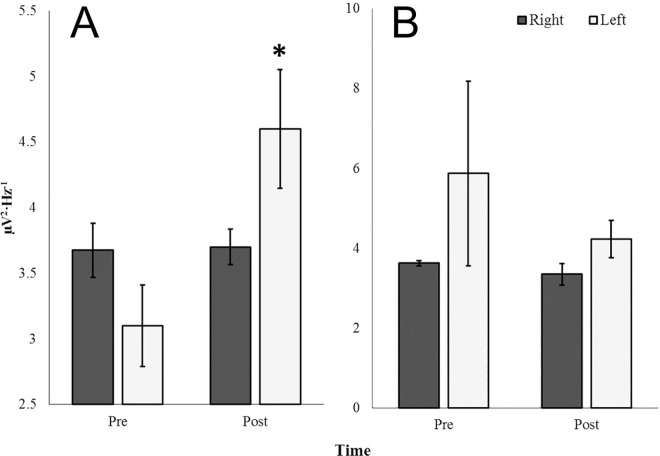
Glutamate Response in a Darkened Environment. Beta2 (20Hz– 30Hz) PDs for periods of pre- and post-injection for right (dark) and left (light) parahippocampal gyri exposed to 1 mL surface injections of 100 nM glutamate (A) and water (B). A significant difference from pre-to-post-injection periods for the left parahippocampal gyrus was revealed (p < .05).

Within a darkened environment, injections of 100 nM glutamate applied to the surface of coronal sections placed in the darkened environment produced increased post-injection (M = 4.60, SEM = .45) beta2 PDs relative to the pre-injection (M = 3.10, SEM = .31) period for the left parahippocampal gyrus [t(6) = 2.73, p < .05, r^2^ = .55 (Fig 16A)] (S6 File). This response was not noted for the right parahippocampal gyrus, nor was it observed when water was applied to the tissue (Fig 16B). The mean photon raw count when the glutamate was applied over the left parahippocampal region was 241.2 per 20 ms and 225.3 per 20 ms when water was applied. The difference (16 photons) per second (50 Hz sampling) was 800 counts. Assuming a typical peak range photon for the equipment to be associated with 4.22·10^−19^ J the increased photon flux density from the tissue after glutamate was applied compared to when only water was applied would have been 3.38·10^−16^ Watts (Joules per s). Because the aperture of the PMT was about 2.25·10^−4^ m^2^, the photon flux power density increase would have been 1.5·10^−12^ W·m^-2^.

The regression equation relating the change in power density for the beta2 band to the numbers of photons was 0.0394 multiplied by the number of photons (plus 4.314, the constant). This means that for an increase of every ~10 photons per s over the left parahippocampal region after glutamate application the corresponding beta2 band power increased by 0.39 μV^2^ ·Hz^-1^. When applied over the 10 Hz increment of beta2 (20 to 30 Hz) this would be 3.9 μV^2^ or about 2 μV.

**Fig 17:**
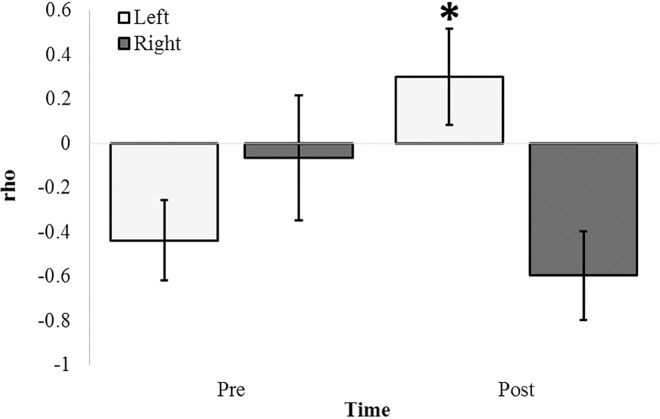
Glutamate Induced Microvolt-Photon Pairings. Non-parametric correlations (Spearman rho values) between beta2 (20Hz– 30Hz) PDs within and raw photon counts over the left (light) and right (dark) parahippocampal gyri for the 25 seconds preceding (Pre) and proceeding (Post) injections of 100 nM glutamate applied to the surface of the coronal sections. Significant differences in non-parametric correlations from pre-to-post-injection periods is noted for the left parahippocampal gyrus (p < .05).

A bivariate non-parametric correlational analysis was performed with beta2 PDs and raw photon counts obtained 10 cm over the tissue within the vertical plane. Spearman rho values indicating the relationship between beta2 and raw photon counts were examined for hemisphere-, compound-, and time-dependent differences. An ANOVA revealed a three-way interaction of these factors [F(1,31) = 8.38, p = .008, η = .23]. The primary source of variance was identified as a difference in rho values associated with the pre-injection period (M = -.44, SEM = .18) and the post-injection period (M = .30, SEM = .20) for the left parahippocampal gyrus [t(6) = 2.61, p < .05, r^2^ = .53]. Fig 17 demonstrates this difference which was not noted for the right parahippocampal gyrus. Alternative PD-photon relationships (e.g. delta PD-photon, theta PD-photon, etc.) were not observed (p>.05).

### Signal Complexity and Frequency Discrimination: Heschl’s Gyrus

**Fig 18:**
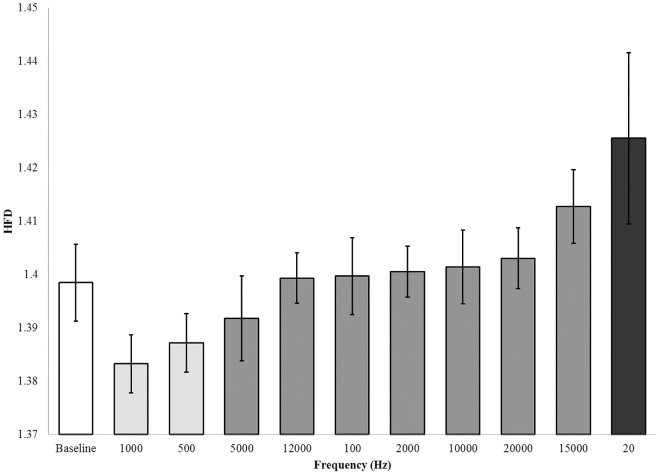
Fractal Dimensions by Frequency: Heschl’s Gyrus. Results of the Higuchi Fractal Dimension (HFD) Kruskall-Wallis test. Groups as revealed by post-hoc Mann-Whitney U tests are indicated by shading of bars, with the exception of the baseline (error bars = SEM).

HFDs were calculated for each transformed segment of EEG data, including the baseline condition, using MatLab. The results of Kolmogorov-Smirnov tests for normalcy were significant, thus non-parametric methodologies were employed. Significant effects for hemisphere (Mann-Whitney U, p = 0.017) and waveform (Mann-Whitney U, p = 0.008), but not location of sensor were found and directed further analyses. Selecting for each individual hemisphere and waveform condition revealed significant differences in the HFDs of the EEG data recorded during the right sine wave condition (KW χ^2^(9) = 17.927, p = 0.036). Subsequent *post-hoc* analyses revealed three groups (average HFDs = 1.38, 1.40, 1.42) where the HFD for 20 Hz (1.425) was largest and different from all other conditions, including baseline (1.398) (Fig 18).

**Fig 19:**
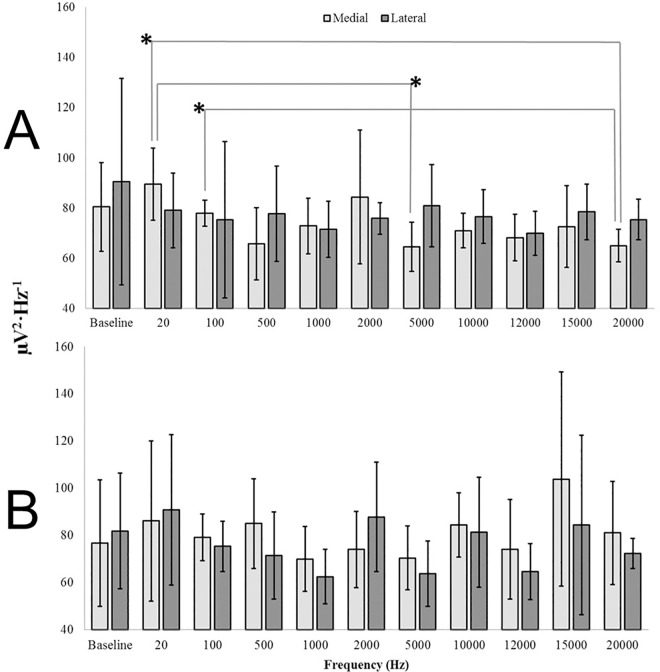
Frequency Discrimination. Theta (4Hz– 7.5Hz) PDs within the postero-medial (Medial; light) and antero-lateral (Lateral; dark) aspect of Heschl’s Gyrus within the left (A) and right (B) hemispheres as a function of square-wave signals with frequencies ranging between 20Hz to 20, 000 Hz. Significant differences after correction (α = .005) are indicated.

ANOVAs revealed that delta (η^2^ = .30), theta (η^2^ = .28), and beta2 (η^2^ = .28) PDs differed as a function of frequency for square-wave stimuli presented to the medial aspect of HG within left hemispheric sagittal sections (p < .05) (S7 File). Differences were not identified as a function of the frequency of the stimulus for all combinations of factors within the right hemisphere, for presentations to the antero-lateral aspect of HG within the left hemisphere, or for sine wave signals (p>.05). It was strictly a square-wave effect. Applying the Bonferonni method, a corrected alpha level (α = .016) was selected as a conservative threshold beyond which differences were considered significant. Frequency-dependent differences in theta band spectral power (4.0 Hz– 7.5 Hz) remained significant after Bonferonni correction (p < .016). Differences within delta and beta2 bands did not meet this threshold. The primary sources of variance were identified to be differences between 20Hz (M = 89.52, SEM = 5.88) and 5,000Hz (M = 64.60, SEM = 4.01), 20 Hz (M = 89.52, SEM = 5.88) and 20,000 Hz (M = 65.02, SEM = 2.64), as well as 100Hz (M = 77.97, SEM = 2.12) and 20,000 Hz (M = 65.02, SEM = 2.64) which are visualized in Fig 19A. The effect sizes (r^2^) associated with each significant difference were .55, .59, and .59 respectively. In contrast, Fig 19B demonstrates the overlap between frequency-responses within the right hemisphere.

A discriminant analysis revealed that wide-band PDs (1.5Hz– 40Hz) derived from the postero-medial aspect of HG within the left hemisphere upon stimulation by square-wave 20Hz and 20,000Hz signals successfully classified 100% of cases in a corrected model [(n = 12), Λ = .09, χ^2^(6) = 16.64, p < .01, canonical R^2^ = .95]. No other combination of factors, including those selecting for the antero-lateral aspect of the same gyrus, could reproduce a successful classification of cases when attempting to discriminate 20Hz and 20,000Hz (p>.05). The same confluence of factors (i.e. postero-medial, left hemisphere, square waves) generated 100% classification of cases when discriminating 20Hz and 5,000Hz, [Λ = .10, χ^2^(6) = 16.27, p < .05, canonical R^2^ = .95]. Together, these results suggest that the posterior and medial aspects of HG within the left hemisphere but not the adjacent anterior and lateral components maintain frequency discrimination capacities long after death in EFA-fixed human brain tissue–a property unobserved within analogous areas of the right hemisphere.

## Discussion

One of the most important perspectives afforded by the pursuit of knowledge through systematic and scientific methods is to assume nothing. Axioms, self-evident truths, and (most frequently) designation by decree of authority or unchallenged faith in traditions have often been major impedances to the types of discoveries that lead to shifts in paradigms and a more accurate or at least a different perspective of the human condition. As neuroscientists we have been taught or have assumed that the fixed human brain is an unresponsive mass of organic residual that has replaced what was once a vital, complex structure that served as the physical substrate for thought, consciousness, and awareness. The results of the present experiments strongly suggest we should at least re-appraise the total validity of that assumption.

Histological analyses indicated that there was general neuronal conservation that is discernable by routine light microscopy (Fig 1). Although neuronal (soma) Nissl-dominant stains do not discern the integrity of the fields of dendrites or the fidelity of their spines, we have found in unpublished studies with rat brains that those fixed in EFA for protracted periods (years) and later processed through modified Fox-Golgi (zinc chromate) methods exhibited some remarkable integrity of dendritic-spine processes. EFA had been selected based upon experimental comparisons as the primary mode of long-term fixation in our laboratory many years ago [15] regardless of the initial post-mortem immersion (for human brains), because of the cytological detail it retained, its compatibility with a multitude of different stain types, and more recently because of its capacity to express immunochemical properties following specific “rejuvenating” pre-treatments.

If some proportion of the living microstructure remains with the potential to be activated, then electrophysiological patterns similar to those in the living brain should be elicited by physiologically-appropriate concentrations of classic neurotransmitters that would influence primarily only particular frequency bands. We selected the parahippocampal region as the primary focus because of the central role of this structure in human cognitive phenomena. First, it is the primary locus for the initial representation of experience (“memory”) as indicated by the marked decrement in this capacity following loss or lesions such as the cases of HM [16] and RB [17]. Secondly, as demonstrated by the precision of Pierre Gloor’s [18] micro- and macro-anatomical analyses this region directly accesses and reciprocally receives input and output respectively from the entire cerebral cortical manifold. Third, this region, particularly in the right hemisphere, is remarkably sensitive in the living state [19] as well as the fixed state [20] to ambient geomagnetic activity to which all human beings are usually immersed.

During the late 19^th^ century portions of the hippocampal region were argued by anti-Darwinian debaters as the unique feature that discriminated human brains (and presumably the special nature of this species) from other primates [21]. It has been known for decades that temporal lobectomies as a treatment for intractable epilepsy eliminated the psychotrophic and hallucinogenic effects of LSD [22]. The central role of the parahippocampal region and its decreased connectivity from the retrosplenial cortex during hallucinatory experiences induced by LSD [23] also highlights the potentially unique feature of this structure. Carhart-Harris et al [23] found that the functional disconnectivity was strongly correlated with the rating of ego “dissolution” that was inferred to reflect the importance of this circuit to maintain the sense of self. Tagliazucchi et al. [24], employing a slightly different approach, noted that the LSD effects enhanced global between-module interactions within those regions rich in 5-HT_2a_ receptors.

Within the fixed dead human brain increases in theta power within the right hippocampal body was observed after application of 100 nM and 100 μM concentrations of serotonin. Unlike the living brain the serotonin immediately and directly apposed the tissue upon application and was not diluted by either the multiple blood-brain barriers or the catabolising environment of enzymes. The double peak suggests two receptor subtypes that are consistent with those reported within the hippocampus [25]. The elicitation of gamma power within an even smaller concentration (10 nm) from the right parahippocampus region suggests an intrinsic separation of some remaining infrastructure that differentiates 4–7 Hz and 30–40 Hz patterns. This is important simultaneity in light of the common observation that gamma ripples are superimposed upon the massive theta activity within this region [26–28]. This intrinsic association has been argued by Bear [29] to be a primary electrophysiological correlate by which consciousness and awareness are coupled to memory. That this structure in the right but not the left hemisphere displayed the effect indicates the responses were specific and that there may be some particular residual within the right hemisphere. Rouleau and Persinger [12] on the bases of similar results have suggested that the implications of the massive historical data base of surgical stimulation of patients and the interpretation of the etiology of their colourful experiences might be reconsidered.

Glutamate is considered the major excitatory neurotransmitter of the brain and is a major correlate of the processes that contribute to long-term potentiation (LTP) which are the first phases of memory consolidation [30]. The peak power density within the gamma range over the left parahippocampal region also suggested that some residual of two receptor subtypes remained with affinities in the nanoMolar and milliMolar range. The increase in power within the gamma range after the applications of these two concentrations was between 0.3 and 0.5 μV^2^·Hz^-1^ which is within the range of shifts in cerebral cortical activity that we have measured to be associated with consciousness and specific tasks [31, 32]. The laterality of the effect was clearly indicated. In contrast the right parahippocampal region displayed power increases that were primarily evident across the gross band of activity; this occurred for the milliMolar range. That the effects were dynamic and not passive was indicated by the transience of the peak response (Fig 9) and the gradual “habituation” or diminishment with repeated trials (Fig 10).

Glutamate has been shown to induce biophotonic activities [33] in neural circuits. Several authors have suggested that biophoton patterns may be central to neural information processing and decoding that may depend upon quantum brain mechanisms [34–36]. The left parahippocampal gyrus responded significantly to surface applications of 100 nM of glutamate solutions by increasing the power spectra within the 20 to 30 Hz range by about 1.5 μV^2^ ·Hz^-1^ compared to the previous baseline conditions while in the darkened environment. This was not observed for the right equivalent region. There were also moderately strong correlations between the numbers of photons emitted after the injection (but not before) and the power density for 30 to 40 Hz, the gamma range but not for other PD frequency bands.

The mean numbers of photons per s was equivalent to a photon flux density of about 10^−12^ W·m^-2^ which is the same order of magnitude as those generated in rat hippocampal slices when coupled to theta activity [37]. This flux density is the same order of magnitude that was measured from the right hemispheres (at the level of the temporal lobe) when people sitting in very dark rooms engaged in vivid imagination about white light compared to mundane thoughts [38]. Finally, the presence of a temporal discrepancy between the left and right temporal lobes for the spectral flux density of photon emissions while human beings sat with their eyes closed in a dark room has been measured for this magnitude [39]. In other words, by simply applying glutamate at concentrations typically encountered within living brain tissue photons were emitted from human tissue that had been fixed in EFA for decades. The flux densities were comparable to that associated with specific cognitions generated by the living brain.

The physical bases to “consciousness” and cognition with the implication of a more ubiquitous property that may occur throughout the universe would be consistent with the philosophy of Spinzoa [40] and the concept of Ernst Mach [41] that the behaviour of any part of the universe (“cosmos”) is determined by all of its parts. Similar, more recent approaches have been expanded and quantified by Hameroff and Penrose [42] and Persinger and St-Pierre [43]. We have operated upon the assumption that either gravity or electromagnetism–or both as these are not mutually exclusive–represent physical candidates which could satisfy these parameters. Our approach has favoured the photon [43] and therefore electromagnetism. The photon may be the fundamental process that relates complex phenomena over large distances of space and time and would be unimpeded by restrictions of speed assuming non-local photon-photon interactions. If this were valid, then an integrating factor must be present such as the commonality of the most dominant constituent, the hydrogen atom and the neutral hydrogen line of 1.42 GHz [44]. It may be relevant (but also potentially spurious) that the average spectral power density produced by the application of glutamate (~2·10^−12^ W·m^-2^ or kg·s^-3^) divided by the change in microvoltage associated with that application (~2·10^−6^ V) results in 10^−6^ A·m^-2^. Applied across the area of the PMT aperture that would be the equivalent of 10^−10^ A associated with the application of the glutamate compared to water. When this current is divided by the unit charge value of 10^−19^ A·s, the residual frequency is 10^9^ Hz or GHz which is well within the range of the neutral hydrogen line. In the absence of a strong hypothetical mechanism which explains how long-deceased biological material could systematically emit photons, this convergence of numbers should be further considered even if with caution.

Systematic injections of different concentrations of two “psychotropic” compounds, nicotine and ketamine, also showed natural, living brain-like responses in terms of both latency and concentration. Again there were anisotropic hemispheric responses within the regions of interest. Enhanced theta power associated with nicotine would be consistent with the memory-enhancing capacity of this cholinergic-stimulating compound [45]. It may be relevant that nicotinamide adenine dinucleotide (NAD), which contains the molecular structure nicotinic acid, is a major source of electrons in living biochemical systems. The purine component of that molecule is synthesized from glutamate, aspartate and glycine. Tryptophan is the precursor of the nicotinamide moiety of NAD and NADP and contributes to the creation of nicotinic acid. From this perspective the similarity of the theta-band enhancement for the right hippocampal regions for both serotonin and nicotine would be expected. These patterns suggest the possibility that a residual of the intrinsic signatures that reflected the complex biochemical reactions within brain tissue may still be present in fixed post-mortem tissue and might be “reactivated”. That the same sites and frequencies were either enhanced by glutamate or suppressed by ketamine at realistic physiological dosages would support this possibility.

Whereas electrophysiological studies are regularly conducted with still-living tissue explants, there are a few notable methodological differences between the aforementioned and what we have presented. First, tissue preparations, whether measured by single electrodes or multi-electrode arrays, are usually no thicker than 1 mm where slices of ~400 μm are typical [46]. Second, measurements of tissue preparations such as those of hippocampal slices are typically conducted within 24 hours of decapitation and within a nutrient-rich medium which is supplemented in various ways to inhibit rapid tissue deterioration [46, 47]. During this period, and despite mitigation efforts, a significant proportion of the cells usually die as inferred by staining procedures [47]. The tissue explants are normally maintained at physiological temperature and immobilized to reduce mechanically-induced damage. Our specimens are chemically fixed, much thicker (> 1cm), older, maintained at room temperature, and not supplemented in any way. It is therefore curious that in both cases, fluctuations in electric potential differences can be observed. Multi-electrode array recordings of tissue explants are known to register spike values of up to 600 μV, though the typical range of fluctuations are within 10–100 μV [46]. Our measurements of post-mortem, fixed tissue have revealed typical fluctuations within 1–80 μV with some high-magnitude transients [12]. In this respect, our measurements are consistent with those observed by others.

Finally, the persistence of essential microstructure was evident by the remaining signal complexity and frequency discrimination that was still apparent within the transverse temporal gyrus. A remarkable frequency dependence for maximum responsivity according to our measures occurred at the lower boundary of the threshold for hearing in the human brain. Compared to baseline measurements, the largest discrepancy occurred around 20 Hz. As recently reviewed by Persinger [48] this is the classic transition between infrasound and regular sound discernment by the human brain. What is less known is that human auditory system does respond to < 20 Hz sound (mechanical vibrations). However, these regions of the system are less expansive and have few afferents to regions of the cortices involved with awareness. This structural substrate appears to remain after death in appropriately fixed brains.

Merker [49] presented an argument that gamma synchrony, rather than representing a cognitively-significant correlate, is more likely an indicator of generic infrastructural control at the level of the tissue. That is, the cognitive correlate of cortical gamma synchrony is really just a necessary co-occurrence rather than a central operator of cognitive states. If one assumes that the brain is “dead” and therefore categorically can’t be conscious, Merker’s [49] interpretation could hold true as we’ve observed a degree of gamma activations which could be indicative of synchrony. However, as self-report methods which require sensory inputs and motor outputs are unavailable to the post-mortem specimens, consciousness and cognitive states cannot be measured without inference by electroencephalography. Therefore, the assumption of an absence of consciousness would be based upon an absence of evidence. From this perspective, if Merker’s interpretation is incorrect and gamma synchrony is in fact cognitively-significant beyond mere activation, the post-mortem brain which displays subtle cortical oscillations, particularly within the theta and gamma bands as demonstrated here, could express some capacity for cognitive activation.

## Supporting Information

S1 File Nicotine Trials. This file contains data used in the analysis involving post-mortem tissue exposed to injections of nicotine.
(XLSX)

S2 File 5-HTP Trials. This file contains data used in the analysis involving post-mortem tissue exposed to injections of 5-HTP.
(XLSX)

S3 File Glutamate. This file contains data used in the analysis involving post-mortem tissue exposed to injections of glutamate.
(XLSX)

S4 File Ketamine Trials. This file contains data used in the analysis involving post-mortem tissue exposed to injections of ketamine.
(XLSX)

S5 File Glutamate with Ketamine Trials. This file contains data used in the analysis involving post-mortem tissue exposed to injections of glutamate with ketamine.
(XLSX)

S6 File Photon Data. This file contains data used in the analysis involving photon counts paired to injections of glutamate.
(XLSX)

S7 File Heschl’s Gyrus. This file contains data used in the analysis involving post-mortem tissue exposed to electrical stimuli directed to the transverse temporal gyri.
(XLSX)

## References

[1] Persinger MA, & Koren SA. A theory of neurophysics and quantum neuroscience: implications for brain function and the limits of consciousness. Int J of Neur. 2007 7 117(2): 157–175. 10.1080/00207450500535784 17365106

[2] Persinger MA, Saroka KS, Koren SA, & St-Pierre LS. The electromagnetic induction of mystical and altered states within the laboratory. J Con Exp Res. 2010 10 1(7): 808–830.

[3] Wijdicks EF. The case against confirmatory tests for determining brain death in adults. Neurology. 2010 7 75(1): 77–83. 10.1212/WNL.0b013e3181e62194 20603486

[4] Wijdicks EF. Brain death worldwide Accepted fact but no global consensus in diagnostic criteria. Neurology. 2002 1 58(1): 20–25. 11781400 10.1212/wnl.58.1.20

[5] Fox CH, Johnson FB, Whiting J, & Roller PP. Formaldehyde fixation. J Histochem Cytochem. 1985 8 33(8): 845–853. 3894502 10.1177/33.8.3894502

[6] Hopwood D. Theoretical and practical aspects of glutaraldehyde fixation. Histochem J. 1972 7 4(4): 267–303. 4118613 10.1007/BF01005005

[7] Kiernan JA. Formaldehyde, formalin, paraformaldehyde and glutaraldehyde: what they are and what they do. Microscopy Today. 2000 12 1(5): 8–12.

[8] Harrison PTC. An ethanol-acetic acid-formol saline fixative for routine use with special application to the fixation of non-perfused rat lung. Laboratory Animals. 1984 10 18(4): 325–331. 6513498 10.1258/002367784780865324

[9] Hasenstaub A, Shu Y, Haider B, Kraushaar U, Duque A, & McCormick DA. Inhibitory postsynaptic potentials carry synchronized frequency information in active cortical networks. Neuron. 2005 8 47(3): 423–435. 10.1016/j.neuron.2005.06.016 16055065

[10] Rivier F, & Clarke S. Cytochrome oxidase, acetylcholinesterase, and NADPH-diaphorase staining in human supratemporal and insular cortex: evidence for multiple auditory areas. Neuroimage. 1997 11 6(4): 288–304. 10.1006/nimg.1997.0304 9417972

[11] Da Costa S, van der Zwaag W, Marques JP, Frackowiak RS, Clarke S, & Saenz M. Human primary auditory cortex follows the shape of Heschl's gyrus. J Neurosci. 2011 10 31(40): 14067–14075. 10.1523/JNEUROSCI.2000-11.2011 21976491 PMC6623669

[12] Rouleau N & Persinger MA. Differential responsiveness of the right parahippocampal region to electrical stimulation in fixed human brains: Implications for historical surgical stimulation studies?. Epi & Behav. 2016 5 60:181–186. 10.1016/j.yebeh.2016.04.028 27208828

[13] Accardo A, Affinito M, Carrozzi M, & Bouquet F. Use of the fractal dimension for the analysis of electroencephalographic time series. Biological cybernetics, 1997 11 77(5): 339–350. 10.1007/s004220050394 9418215

[14] Newcomer JW, Farber NB, Jevtovic-Todorovic V, Selke G, Melson AK, Hershey T et al Ketamine-induced NMDA receptor hypofunction as a model of memory impairment and psychosis. Neuropsychopharmacology, 1999 6 20(2): 106–118. 10.1016/S0893-133X(98)00067-0 9885791

[15] Persinger MA. Brain mast cell numbers in the albino rat: sources of variability. Behav Neur Bio. 1979 3 25(3): 380–386. 10.1016/s0163-1047(79)90448-5 380555

[16] Scoville WB, & Milner B. Loss of recent memory after bilateral hippocampal lesions. J Neuro Neurosurg Psych. 1957 2 20(1): 11–21. 10.1136/jnnp.20.1.11 PMC497229 13406589

[17] Zola-Morgan S, Squire LR, & Amaral DG. Human amnesia and the medial temporal region: enduring memory impairment following a bilateral lesion limited to field CA1 of the hippocampus. J Neuro, 1986 10 6(10): 2950–2967. 10.1523/JNEUROSCI.06-10-02950.1986 PMC6568782 3760943

[18] Gloor P. The temporal lobe and limbic system. Oxford University Press, USA 1997.

[19] Saroka KS, Caswell JM, Lapointe A, & Persinger MA. Greater electroencephalographic coherence between left and right temporal lobe structures during increased geomagnetic activity. Neuro Let. 2014 2 560: 126–130. 10.1016/j.neulet.2013.11.024 24287380

[20] Costa J, Rouleau N, & Persinger MA. Differential spontaneous photon emissions from cerebral hemispheres of fixed human brains: Asymmetric coupling to geomagnetic activity and potentials for examining post-mortem intrinsic photon information. Neuro Med. 2016 6 7(2): 49–59.

[21] Jensen JV. Return to the Wilberforce–Huxley Debate. The British Journal for the History of Science. 1988 6 21(02): 161–179.

[22] Serafetinides EA. The significance of the temporal lobes and of hemispheric dominance in the production of the LSD-25 symptomatology in man: a study of epileptic patients before and after temporal lobectomy. Neuropsychologia. 1965 3 3(1): 69–79.

[23] Carhart-Harris RL, Muthukumaraswamy S, Roseman L, Kaelen M, Droog W, Murphy K et al Neural correlates of the LSD experience revealed by multimodal neuroimaging. Proc Nat Ac Sci, 2016 3 113(17): 4853–4858. 10.1073/pnas.1518377113 PMC4855588 27071089

[24] Tagliazucchi E, Roseman L, Kaelen M, Orban C, Muthukumaraswamy SD, Murphy K et al Increased global functional connectivity correlates with LSD-Induced ego dissolution. Cur Bio. 2016 4 26(8): 1043–1050. 10.1016/j.cub.2016.02.010 27085214

[25] Pazos A, Gonzalez AM, Waeber C, & Palacios JM. Multiple Serotonin Receptors in the Human Brain. In Receptors in the human nervous system. Mendelsohn F. A., & Paxinos G. (Eds.). Academic Press, Inc 1991 74–95.

[26] Belluscio MA, Mizuseki K, Schmidt R, Kempter R, & Buzsáki G. Cross-frequency phase–phase coupling between theta and gamma oscillations in the hippocampus. J Neuro. 2012 1 32(2): 423–435. 10.1523/JNEUROSCI.4122-11.2012 PMC3293373 22238079

[27] Quilichini P, Sirota A, & Buzsáki G. Intrinsic circuit organization and theta–gamma oscillation dynamics in the entorhinal cortex of the rat. J of Neuro. 2010 Aug 30(33): 11128–11142. 10.1523/JNEUROSCI.1327-10.2010 PMC2937273 20720120

[28] Sirota A, Montgomery S, Fujisawa S, Isomura Y, Zugaro M, & Buzsáki G. Entrainment of neocortical neurons and gamma oscillations by the hippocampal theta rhythm. Neuron. 2008 11 60(4): 683–697. 10.1016/j.neuron.2008.09.014 19038224 PMC2640228

[29] Bear MF. A synaptic basis for memory storage in the cerebral cortex. Proc Nat Acad Sci. 1996 11 93(24): 13453–13459. 8942956 10.1073/pnas.93.24.13453 PMC33630

[30] Bashir ZI, Bortolotto ZA, Davies CH, Berretta N, Irving AJ, Seal AJ et al Induction of LTP in the hippocampus needs synaptic activation of glutamate metabotropic receptors. Nature. 1993 5 363: 347–350. 10.1038/363347a0 8388549

[31] Persinger MA & ¬¬¬Saroka KS. Human quantitative electroencephalographic and Schumann Resonance exhibit real time coherence of spectral densities: implications for interactive information processing. J Sig Inf Pro. 2015 5 6: 153–164.

[32] Saroka KS, Vares DE, & Persinger MA. Similar Spectral Power Densities Within the Schumann Resonance and a Large Population of Quantitative Electroencephalographic Profiles: Supportive Evidence for Koenig and Pobachenko. PloS one. 2016 Jan 11(1): 1–22. 10.1371/journal.pone.0146595 PMC4718669 26785376

[33] Tang R. & Dai J. Biophoton signal transmission and processing in the brain. J Photochem Photobio B: Bio. 2014 10 139: 71–75. 10.1016/j.jphotobiol.2013.12.008 24461927

[34] Baars BJ, & Edelman DB. Consciousness, biology and quantum hypotheses. Phys Life Rev. 2012 9 9(3), 285–294. 10.1016/j.plrev.2012.07.001 22925839

[35] Persinger MA, Dotta BT, & Saroka KS. Bright light transmits through the brain: Measurement of photon emissions and frequency-dependent modulation of spectral electroencephalographic power. World Journal of Neuroscience. 2013 12 3(1): 10–16.

[36] Tegmark M. Importance of quantum decoherence in brain processes. Phys Rev E. 2000 4 61(4): 4194–4206. 10.1103/physreve.61.4194 11088215

[37] Kobayashi M, Takeda M, Sato T, Yamazaki Y, Kaneko K, Ito K et al In vivo imaging of spontaneous ultraweak photon emission from a rat’s brain correlated with cerebral energy metabolism and oxidative stress. Neuro Res. 1999 7 34(2): 103–113. 10.1016/s0168-0102(99)00040-1 10498336

[38] Dotta BT, Saroka KS, & Persinger MA. Increased photon emission from the head while imagining light in the dark is correlated with changes in electroencephalographic power: Support for Bókkon's Biophoton Hypothesis. Neuro Let. 2012 4 513(2): 151–154. 10.1016/j.neulet.2012.02.021 22343311

[39] Dotta BT, & Persinger MA. Increased photon emissions from the right but not the left hemisphere while imagining white light in the dark: The potential connection between consciousness and cerebral light. J Con Exp Res. 2011 12 2(10): 1463–1473.

[40] Grene, MG. Spinoza: a collection of critical essays. 1978

[41] Mach, E. The Science of Mechanics; a Critical and Historical Account of Its Development, by Dr. Ernst Mach. Tr. from the German by Thomas J. McCormack. With 250 Cuts and Illustrations. Chicago, The Open court publishing Company. 1919.

[42] Hameroff S, & Penrose R. Consciousness in the universe: A review of the ‘Orch OR’theory. Phys Life Rev. 2014 3 11(1), 39–78. 10.1016/j.plrev.2013.08.002 24070914

[43] Persinger MA, St-Pierre LS. The physical bases to consciousness: Implications of convergent quantifications. J. Sys. Integrat. Neurosci. 2015 11 1: 55–64.

[44] Hummel E, Dettmar RJ, & Wielebinski R. Neutral hydrogen and radio continuum observations of NGC 55. Astron Astrophys. 1986 9 166: 97–106.

[45] Huerta PT, & Lisman JE. Bidirectional synaptic plasticity induced by a single burst during cholinergic theta oscillation in CA1 in vitro. Neuron. 1995 11 15(5): 1053–1063. 7576649 10.1016/0896-6273(95)90094-2

[46] Oka H, Shimono K, Ogawa R, Sugihara H, & Taketani M. A new planar multielectrode array for extracellular recording: application to hippocampal acute slice. Journal of neuroscience methods. 1999 10 93(1): 61–67. 10598865 10.1016/s0165-0270(99)00113-2

[47] Egert U, Schlosshauer B, Fennrich S, Nisch W, Fejtl M, Knott T,. . . & Hämmerle H. A novel organotypic long-term culture of the rat hippocampus on substrate-integrated multielectrode arrays. Brain Research Protocols. 1998 6 (4): 229–242. 9630647 10.1016/s1385-299x(98)00013-0

[48] Persinger MA. Infrasound, human health, and adaptation: an integrative overview of recondite hazards in a complex environment. Natural Hazards. 2014 8 70(1): 501–525.

[49] Merker B. Cortical gamma oscillations: the functional key is activation, not cognition. Neuroscience & Biobehavioral Reviews, 2013 3 37(3): 401–417. 23333264 10.1016/j.neubiorev.2013.01.013

