# Correction: When Is the Brain Dead? Living-Like Electrophysiological Responses and Photon Emissions from Applications of Neurotransmitters in Fixed Post-Mortem Human Brains

**DOI:** 10.1371/journal.pone.0169378

**Published:** 2016-12-28

**Authors:** The PLOS ONE Staff

## Notice of Republication

This article was republished on December 15, 2016, to correct an error in the author order that was introduced during the typesetting process. The publisher apologizes for the error. Please download this article again to view the correct version. The originally published, uncorrected article and the republished, corrected article are provided here for reference.

## Supporting Information

S1 File Originally published, uncorrected article. (PDF)

S2 File Republished, corrected article. (PDF)
